# Challenges in confirming the position of a central venous catheter in the presence of an arterio‐venous haemodialysis fistula

**DOI:** 10.1002/anr3.12264

**Published:** 2023-11-28

**Authors:** C. R. Evans, T. M. Hall

**Affiliations:** ^1^ Department of Paediatric Intensive Care St Georges Hospital London UK; ^2^ Department of Anaesthesia St Georges Hospital London UK

**Keywords:** arteriovenous fistula, central venous catheter, chronic renal failure, intensive care unit

A 71‐year‐old man was admitted to the Cardiothoracic Intensive Care Unit following implantation of a left ventricular assist device (LVAD) (Impella, Abiomed, Danvers, MA, USA) and percutaneous coronary intervention (PCI) procedure. His medical history included coronary artery disease and end‐stage chronic kidney disease requiring haemodialysis via an arteriovenous (AV) fistula on his right arm. He was transferred to our centre with new onset heart failure and was found to have a left ventricular ejection fraction of 22% on transthoracic echo. Percutaneous coronary intervention was deemed too high risk to undertake without LVAD support. During a protracted recovery, on day 46, he required a replacement central venous catheter (CVC) and dialysis catheter. The left internal jugular vein was chosen due to the presence of existing vascular access devices elsewhere. An 8.5 Fr, 20 cm quad‐lumen CVC (Multicath 4expert, Vygon, Aachen, Germany) and a 13.5 Fr, 20 cm dual lumen dialysis catheter (Hemo‐cath, Nikkiso Co Ltd, Tokyo, Japan) were sited at a depth of 18 cm and 17 cm respectively, with the dialysis catheter placed proximally.

**Figure 1:**
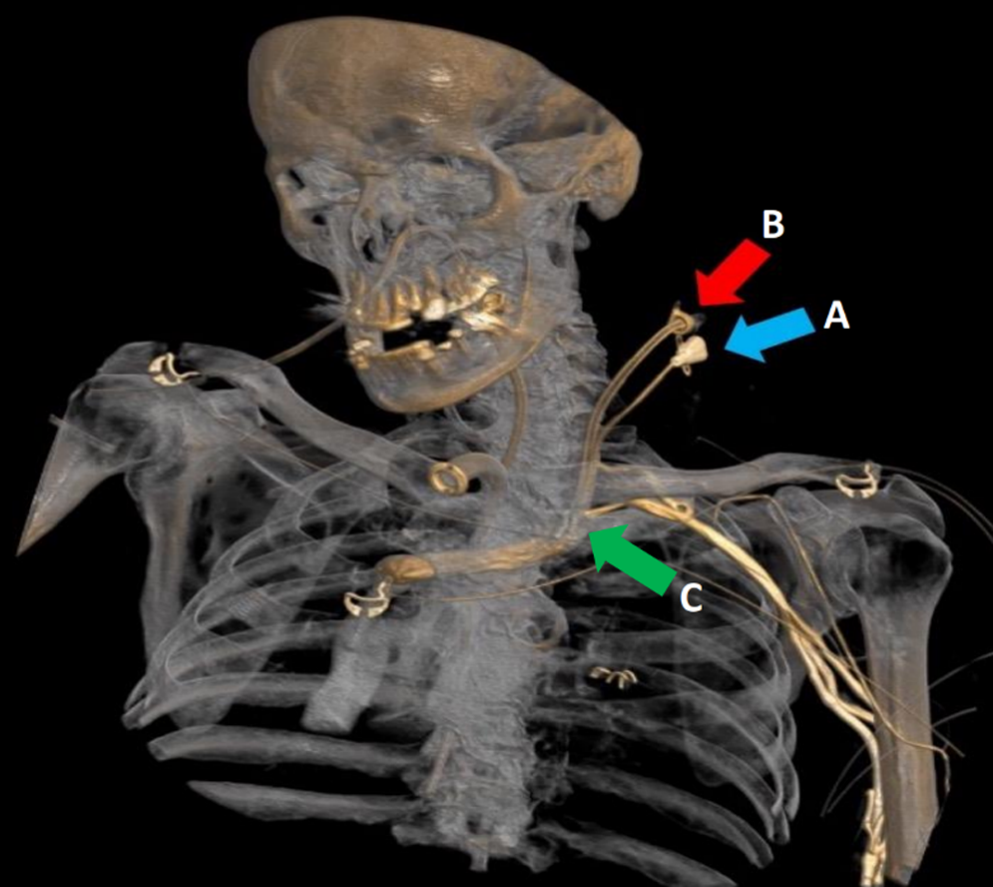
Reconstructed 3D image of CT angiogram of the aortic arch and carotid arteries. The CVC (A) and dialysis catheter (B) can be seen entering the left aspect of the neck and the left internal jugular vein. The CT report confirmed the presence of both catheter tips in the distal left brachiocephalic vein (C). Contrast is noted to obscure the distal part of both catheters in this image.

Blood gas analysis from the distal lumen of the new CVC showed a pO_2_ of 10.8 kPa (F_I_O_2_ of 0.28). A contemporaneous arterial line sample indicated an arterial pO_2_ of 10.5 kPa. A sample from the distal lumen of the dialysis catheter indicated a more reassuring pO_2_ of 4.24 kPa. Repeat CVC samples showed a pO_2_ of 10 kPa from the distal lumen whilst samples taken from proximal CVC lumens indicated a pO_2_ of 4.62 kPa, consistent with venous results. Because of these results, we were concerned that the CVC had punctured the left carotid artery. Neither line was transduced at this stage and a computed tomography (CT) angiogram was arranged urgently, which confirmed an appropriate position for both lines (Fig. 1).

This case highlights the difficulty of interpreting blood gas samples taken from a CVC in a patient with an AV fistula. The presence of a high pO_2_ cannot be interpreted accurately because of abnormal flow of arterial blood from the fistula. However, results compatible with venous samples were taken from the proximal lumens of the CVC which complicated the interpretation of results. Although rare, cases of patients with AV fistulae in whom CVC location is unclear due to unexpected blood gas analysis data have been previously reported [1, 2]. It is well established that the central veins demonstrate laminar flow and that laminae vary in their oxygenation, indicating that mixing of content between the laminae does not necessarily occur [3]. It seems most likely in our case that the distal CVC lumen was situated sufficiently close to the fistula to allow aspiration from an arterial, well‐oxygenated stream of blood.

As per Association of Anaesthetists guidance [4], pressure transduction is an option for assessing placement of a CVC, alongside blood gas assessment. Manometer tubing can be attached to the cannula used in the modified Seldinger technique to allow the confirmation of venous placement before dilation [5], although care must be taken to maintain sterility. Transducing all CVC lumens would have helped confirm intravenous placement in this case. However, we highlight the importance of further investigation of CVC placement prior to use in cases where malposition cannot be ruled out.
